# Inactivation of *RB1* , *CDKN2A* , and *TP53* have distinct effects on genomic stability at side‐by‐side comparison in karyotypically normal cells

**DOI:** 10.1002/gcc.23096

**Published:** 2022-09-30

**Authors:** Natalie Andersson, Karim H. Saba, Linda Magnusson, Jenny Nilsson, Jenny Karlsson, Karolin H. Nord, David Gisselsson

**Affiliations:** ^1^ Division of Clinical Genetics, Department of Laboratory Medicine Lund University Lund Sweden; ^2^ Division of Oncology‐Pathology, Department of Clinical Sciences Lund University Lund Sweden; ^3^ Clinical Genetics and Pathology, Laboratory Medicine Lund University Hospital, Skåne Healthcare Region Lund Sweden

**Keywords:** CDKN2A, chromosomal instability, phylogeny, RB1, TP53, tumor suppressor

## Abstract

Chromosomal instability is a common feature in malignant tumors. Previous studies have indicated that inactivation of the classical tumor suppressor genes *RB1*, *CDKN2A*, and *TP53* may contribute to chromosomal aberrations in cancer by disrupting different aspects of the cell cycle and DNA damage checkpoint machinery. We performed a side‐by‐side comparison of how inactivation of each of these genes affected chromosomal stability in vitro. Using CRISPR‐Cas9 technology, *RB1*, *CDKN2A*, and *TP53* were independently knocked out in karyotypically normal immortalized cells, after which these cells were followed over time. Bulk RNA sequencing revealed a distinct phenotype with upregulation of pathways related to cell cycle control and proliferation in all three knockouts. Surprisingly, the *RB1* and *CDKN2A* knocked out cell lines did not harbor more copy number aberrations than wild‐type cells, despite culturing for months. The *TP53*‐knocked out cells, in contrast, showed a massive amount of copy number alterations and saltatory evolution through whole genome duplication. This side‐by‐side comparison indicated that the effects on chromosomal stability from inactivation of *RB1* and *CDKN2A* are negligible compared to inactivation of *TP53*, under the same conditions in a nonstressful environment, even though partly overlapping regulatory pathways are affected. Our data suggest that loss of RB1 and CDKN2A alone is not enough to trigger surviving detectable aneuploid clones while inactivation of TP53 on its own caused massive CIN leading to saltatory clonal evolution in vitro and clonal selection.

## 1 INTRODUCTION

Aneuploidy and segmental copy number aberrations are common features in tumors, especially high‐grade adult carcinomas and many childhood cancers.^1^, ^2^, ^3^, ^4^ In addition, chromosomal instability (CIN), that is, an increased rate of chromosome missegregation,^5^ is often observed, in these cancers, further contributing to aneuploidisation. Mechanisms causing CIN include defects in chromosome cohesion, the spindle assembly checkpoint (SAC), kinetochore–microtubule attachment, cell‐cycle regulation, and an increased number of centrosomes (inducing merotely).^6^, ^7^, ^8^ Proteins regulating the cell‐cycle have the capacity to halt cells with DNA damage and/or missegregated chromosomes from cell cycle progression. Inactivation of the corresponding genes may, consequently, permit proliferation of nondiploid cells. Mutations in and deletions of chromosomal regions encompassing tumor suppressor genes such as *RB1*, *CDKN2A*, and *TP53*, essential for cell cycle control in eukaryotic cells, are commonly found across several types of neoplasia.^2^, ^9^, ^10^, ^11^

*RB1* is a tumor suppressor whose homozygous inactivation catalyzes development of the rare tumor retinoblastoma.^12^, ^13^ Its protein product pRb is a constituent of the G1/S cell cycle checkpoint, hindering progression to S‐phase in presence of faulty double strand break repair caused by defective canonical nonhomologous end joining (cNHEJ).^14^ Activated pRb physically binds to the E2F‐DP heterodimer protein and remodulates chromatin, resulting in an inhibition of E2F‐DP activity. Non pRb bound E2F‐DP activates cyclins, cyclin dependent kinases and *PCNA*, aiding the transition from G1 to S‐phase. The protein pRb also inhibits the production of cyclin E as well as MAD2. Conversely, loss of pRb results in an overexpression of MAD2, which has been shown to induce CIN.^15^, ^16^ Loss of pRb has previously been shown to increase chromosomal instability and cause aneuploidy.^16^, ^17^, ^18^, ^19^

*CDKN2A* encodes two different proteins: p14/ARF and p16/INK4a. The protein p14/ARF can downregulate E2F‐dependent transcription, causing G1/S arrest. It also inhibits MDM2, which controls the activity and stability of p53. Loss of p14/ARF hence has a similar effect as loss of p53, that is, abrogation of cell cycle arrest in G2, leading to apoptosis. The protein p16/INK4a binds to CDK4/6 and inhibits its ability to phosphorylate pRb, keeping pRb bound to E2F1 and causing G1/S arrest. *CDKN2A* dysregulation has been shown to cause aneuploidy and CIN.^20^ Both loss of p14/ARF and p16/INK4a may generate an increased incidence of aneuploidy and supernumerary centrosomes through centriole pair splitting, which in turn drive aneuploidy through unequal segregation of the genomic material during mitosis.^21^, ^22^ However, the evidence so far for *CDKN2A* and *RB1*, respectively causing aneuploidy and CIN, is modest and merely performed with older cytogenetic techniques such as metaphase spreads. In addition, many of the cell lines used are known to be genetically unstable in themselves.^23^, ^24^

*TP53* is a tumor suppressor encoding the protein p53 involved in pathways encompassing hundreds of genes, acting as a response to a variety of stress signals, inducing apoptosis, cellular senescence, or cell cycle arrest. If the stress is removed, p53 causes an upregulation of *MDM2* and thereby induces its own degradation, resulting in a half‐life between 5 and 20 min. p53 loss of function may facilitate aneuploidy and enable cells to survive otherwise lethal chromosomal imbalances.^25^, ^26^ There is, however, some evidence that loss of p53 by itself may not be a primary cause of aneuploidy, but may synergize with other alterations to promote aneuploidy and facilitate chromosomal imbalances through indirect mechanisms.^27^ *TP53*‐alterations are often accompanied by other genetic alterations and seen late in the evolution of a tumor, in which case aneuploidy is already present,^5^ but this may vary across cancers.

In this study, we sought to disentangle the effect the three classical tumor suppressor genes *RB1*, *CDKN2A* and *TP53* has on chromosomal instability. Karyotypically normal hTERT immortalized human fibroblasts were subjected to CRISPR‐Cas9 mediated knock‐out of *RB1*, *CDKN2A*, and *TP53*, respectively, and the resulting clones were cultured and analyzed under close to identical conditions. In addition, a dataset of prolonged passaging of the wild type cell line was analyzed for comparison to its intrinsic rate of CIN. High‐resolution copy number profiling and bulk RNA sequencing was performed at multiple passaging times for each cell line. Our data suggest that loss of *RB1* and *CDKN2A* alone is not enough to trigger surviving detectable aneuploid clones while inactivation of *TP53* on its own caused massive CIN leading to saltatory clonal evolution in vitro and clonal selection.

## 2 RESULTS

### 2.1 Clonal evolution under prolonged passaging

**FIGURE 1:**
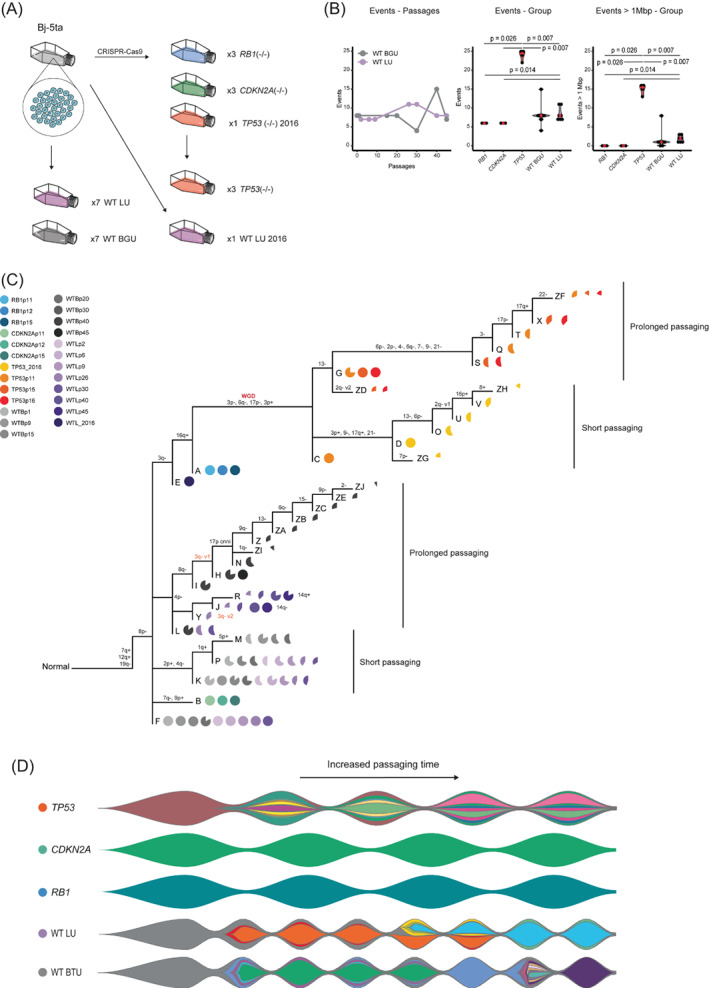
Experimental setup and copy number analysis (A) The experimental setup. Wild type Bj‐5ta cells were cultured for 45 passages and samples for SNP‐array were extracted at 7 time points. The experiment was repeated at two different laboratories, one at Lund University (LU) and one at Ben Gurion University (BGU).^29^ CRISPR‐technique was used to knock out *RB1*, *CDKN2A*, and *TP53* in three different cell populations. Samples for SNP‐array and RNA‐seq were extracted at three different passages. One wild type sample and one *TP53* knocked‐out cell line from 2016 from which one single sample was available were included. (B) The number of genetic alterations (Events) as a function of passages for the cells cultured at LU and BGU. The middle graph illustrates the total number of genetic alterations found in each sample and the rightmost graph the number of events larger than 1 Mbp across the samples. Significant differences are annotated (Dataset S1). (C) Phylogenetic subclone tree based on the SNP‐array data for all samples (Dataset S1). The legend indicates the included samples and their corresponding color code. Darker colors imply a higher passaging number. Pie charts illustrate the proportion of cells in a particular sample that has a specific set of genetic alterations. The chromosomal alterations encompassed by a specific branch are shown above the branches. (D) Fishplots for the *TP53*, *RB1*, and *CDKN2A*‐knocked‐out cell lines as well as the WT cells cultured at either LU or BGU (Dataset S1)

The cell line Bj‐5ta consists of fibroblasts with a normal karyotype and is known to lack tumorigenic characteristics.^28^ The cells have been transfected with an hTERT‐expressing plasmid resulting in a constantly active telomerase, continuously sustaining its telomeres, thus allowing Bj‐5ta cells to proliferate for a prolonged time compared to normal cells. Few studies have analyzed the genetic profile of this cell line after passaging beyond the Hayflick limit, restraining cells with regular cellular senescence. In a previous study, Bj‐5ta cells were subcultured for a total of 45 passages at two different laboratories (Figure 1A,B).^29^ That study illuminated the retained evolutionary capacity of Bj‐5ta, as the initial clone was replaced with a new, genetically distinct, subclone after approximately 20 passages.^29^ This type of inherent clonal replacement in Bj‐5ta was confirmed in our study through phylogenetic analysis of the same original dataset (Dataset S1, Figure 1C,D, and S1). Prolonged culturing by passaging the cells more than 20 times resulted in a significant bottleneck. This baseline evolutionary capacity of Bj‐5ta was taken into account in the following interpretation of results from Bj‐5ta.

### 2.2 CRISPR‐mediated knock‐out of *CDKN2A* and *RB1* results in few copy number alterations

CRISPR was used to knock out *CDKN2A*, *RB1* and *TP53* in three different Bj‐5ta cell populations. Cells were collected at three time points within the stable interval of the cell line (before 20 weeks of culture). Most detected genetic alterations were small structural changes, except in the *TP53* knocked‐out cells that showed a significantly elevated number of larger chromosomal aberrations (≥1 Mbp) compared to all other groups. The *RB1* and *CDKN2A* knocked‐out cell lines were diploid and, interestingly, showed very few chromosomal aberrations and even significantly less genetic alterations than one of the WT strains (Figure 1B, Dataset S1).

The *CDKN2A* knocked‐out cells showed merely two unique alterations, namely a 7q deletion (7q‐) resulting in an intragenic loss of *MAGI2*, a gene reported as altered in many tumor types,^30^, ^31^ and a gain in 9p involving half of *CNTLN*, a gene involved in centriole–centriole cohesion and protein localization,^32^ forming Subclone B detected in all cells at all passages (Figure 1C). Similarly, the *RB1* knocked‐out cells showed only two genetic alterations, 3q− (*RSRC1*, intragenic) which is shared with one of the WT samples, and 16q+ (*WWOX*, intragenic), both of which are shared with the *TP53* knocked‐out cells (subclone A). Surprisingly little aneuploidy was seen (Figure S2A,B). This indicates that loss of *CDKN2A* or *RB1* alone is not sufficient to promote the formation of aneuploid clonal cell populations.

A complex evolutionary pattern was seen in the *TP53* knocked‐out cells compared to the *RB1* and *CDKN2A* knocked‐out cells, respectively (Figure 1C, Figure S2C and S3a). All *TP53*‐knocked cells exhibited a whole genome duplication, also confirmed by G‐banding (Figure S3b). They additionally exhibited multiple other chromosomal aberrations as well as a complex alteration in the centromeric part of 6q (Figure S3C). Between the first and second time point for analysis, an entirely new branch appeared, containing multiple additional chromosomal aberrations. This was in stark contrast to both the *RB1* and *CDKN2A* knocked‐out cell lines, that showed no capability to generate new surviving detectable clones over time (Figure S4).

### 2.3 RNA sequencing reveals distinct phenotypical representations

**FIGURE 2:**
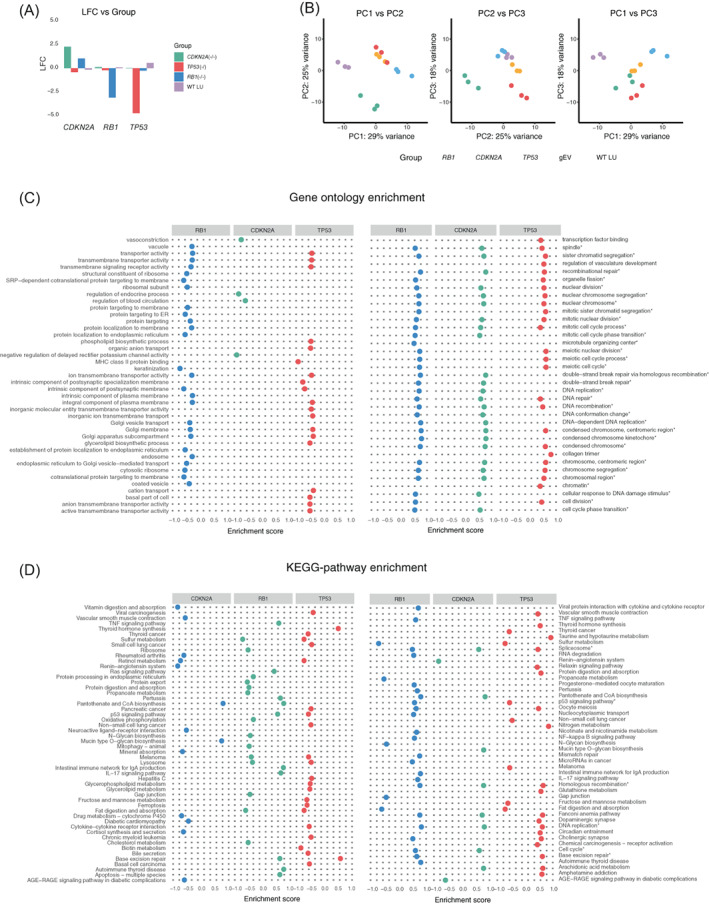
RNA and enrichment analysis. (A) The log fold change (LFC; *y*‐axis) for the knocked‐out genes (legend) across samples (*x*‐axis). (B) PCA plot based on regularized log (rlog) transformed raw RNA‐seq counts. The three first principal components are shown in this graph. (C) The top 20 suppressed (left) and activated (right) GO‐terms present in at least one group. (D) The top 20 suppressed (left) and activated (right) GO‐ and KEGG‐pathways present in at least one group. The asterisks (*) in C and D indicate GO‐terms and KEGG‐pathways mentioned in the text

To verify the phenotype of the respective knockouts, RNA sequencing was performed followed by differential expression analysis (Dataset S2, Figure S5a). The expressions levels of *RB1* and *TP53* were low in the corresponding knocked out cell lines, compared to the empty vector samples (Figure 2A). The *CDKN2A* knock‐outs showed a high expression level of the mutated transcript, likely as a compensatory mechanism. Principal component analysis of regularized log transformed counts for each gene in each sample revealed a clear in‐group clustering (Figure 2B), indicating a similar phenotypical representation. There was no clear trajectory as a function of passaging time. For the *TP53* knocked‐out cells we could see a tendency for the samples to follow a trajectory, suggesting that they were still under development in culture (Figure S5B).

The complete gene lists obtained from the DESeq2 analysis was used as input to the ClusterProfiler functions gseaGO and gseaKEGG to assess significant suppression or activation of genes linked to specific GO‐terms or KEGG‐pathways. For the WT samples there were no significant GO‐terms and the KEGG‐pathways were nonspecific and encompassed few genes (Dataset S3). Among GO‐terms, the *RB1*, *CDKN2A* and *TP53* knocked‐out cell lines all showed upregulation of genes related to cell cycle processes, cell division and chromosome segregation (Figure 2C,D). In all knocked‐out cell lines, DNA replication pathways were significantly upregulated. For KEGG pathways, upregulation was seen in pathways related to cell cycle, DNA replication and the spliceosome. As expected, the p53 signaling pathway was downregulated in the *TP53*‐knocked out cells. Hence, despite not showing evidence of CIN, the *RB1* and *CDKN2A* knocked‐out cells exhibited a phenotype at RNA‐level consistent with inactivation of cell cycle checkpoints.

## 3 DISCUSSION

It has previously been assumed that depletion of *CDKN2A* or *RB1* induces CIN in proliferating human cells. However, when knocking out each of these genes individually in karyotypically normal immortalized cells using CRISPR‐technique in the present study, no detectable aneuploidy or other signs of CIN were seen using high resolution whole genome genotyping arrays. This despite the cells being cultured for months, making remaining wild type protein products unlikely. Hence, our study questions the notion that loss of *RB1* or *CDKN2A* alone gives rise to clonal expansions of aneuploid cell populations. In sharp contrast, the *TP53* knocked‐out cell line exhibited whole genome duplications in all cells analyzed, indicating that cells harboring a doubling of the entire genome have an increased fitness compared to diploid cells in the absence of *TP53*. These near‐tetraploid cells might be more resilient to losses and gains of chromosomes compared to a diploid cell by having a larger reserve capacity of chromosomal copies. This is further strengthened by the observed expansion of multiple clones with additional losses and gains of chromosomal segments following the whole genome duplication, indicative of a form of aneuploidy tolerance in *TP53* defective cells, previously suggested by multiple studies.^33^, ^34^, ^35^ It may also suggest that loss of *CDKN2A* or *RB1* does not, alone, allow this tolerance, explaining the absence of surviving larger clones in this experiment, possibly due to a still active p53‐system in these cells.

Previous studies have shown that depletion of *CDKN2A* and *RB1* result in extra centrosomes, causing CIN. However, an extra number of centrosomes does not necessarily mean that the cells are bound for aneuploidy. In cells with intact p53 function, multipolar cell division is often avoided by clustering of extra centrosomes.^36^ Cells with extra centrosomes undergoing bipolar division can sustain aneuploidy by merotelic attachments causing lagging chromosomes.^8^, ^37^ Still, the generation of aneuploidy at cell division does not necessarily mean that we obtain surviving cells with clonal chromosome alterations, since the aneuploid cell have to go into the cell cycle again and be allowed to pass through it, that is, a certain tolerance to aneuploidy is needed.

Acquired copy number alterations may alter the expression of the genes in the affected segment, pushing the cell into a new phenotypical state. Eventually, this will either generate a significant disturbance of vital functions resulting in cell death or it might adapt to the changes by epigenomic alterations resulting in the cell entering a new stable state which allows it to enter the cell cycle again. This state might either result in the cell getting outcompeted or getting an increased fitness compared to other cells in the area, thus resulting in clonal expansion. For the latter to occur, a cell with copy number alterations must maintain the qualities allowing it to pass through the cell cycle, if yet a modified such. An explanation, based on this rationale, for why we do not see an increase in copy number aberrations in *RB1*‐ or *CDKN2A*‐depleted cells in our experimental setup, could be that these cells do not manage to generate descendants with chromosomal imbalances that can enter and pass through the entire cell cycle again. If there is an underlying high level of CIN, resulting in nonclonal, random defects, they are not measurable through array analyses which typically requires a clone size >10% to be detectable. Hence, we do not reject the hypothesis of CIN resulting from *RB1* or *CDKN2A* loss in our experimental setup, but we can conclude that there is a major difference between how cells lacking either *RB1*, *CDKN2A* or *TP53* manifest as new clones. But then, what makes loss of *TP53* different from *RB1* and *CDKN2A* in the sense of copy number alterations?

It has previously been shown that defective *TP53* affects the function of homologous recombination repair and nonhomologous end joining. Hence the mending of double strand breaks will be deficit, resulting in an increased incidence of structural chromosome changes.^38^ Neither *RB1* nor *CDKN2A* seem to have a strong scientifically proven connection to defects in these reparation mechanisms. Notably, the G1/S and G2/M checkpoints do not react to copy number imbalances per se, but merely DNA‐damage such as single strand breaks, double strand breaks, oxidations, alkylations, deaminations and mismatches. Many copy number aberrations, such as intrachromosomal aberrations or copy number alterations affecting entire chromosomes or arms, will not be halted at these checkpoints. Hence, they are allowed to continue to the spindle assembly checkpoint (SAC). Also, the SAC has been shown to be affected by loss of *TP53*,^39^ but the effect may vary between cell types.^26^ Consequently, aneuploid p53 defective cells are allowed to continue the cell cycle, which increases the risk of missegregation and further aneuploidy.^39^ Neither *RB1* nor *CDKN2A* seem to have an effect on the SAC. Additionally, the SAC does not normally react upon intrachromosomal aberrations, unless they make it hard to align the chromosomes before anaphase, through defects affecting the centromeres, for example, prolonging its activity compared to normal cells. It does however react to aneuploidy. Whether a p53‐defect induces aneuploidy through creating a defective mitotic process or by simply allowing proliferation of cells with deviating chromosomal status is unclear, and additional studies are needed to sort out this conundrum. Also, even if the pathways of *CDKN2A* and *RB1* have been altered, cell cycle arrest and apoptosis can still be initiated in these knocked‐out cells through activation of p53‐pathways, further impeding the generation of surviving clones. *TP53* loss might thus be a prerequisite for cells to survive CIN and go on to generate clonal populations,^35^ besides contributing to triggering CIN by itself.

A possible risk with our experimental setup is that protein products are still prevailing in the cell due to slow degradation or that the gene is not adequately knocked out. When performing CRISPR‐Cas9 mediated knockout, thorough assessment of the actual knock‐out or depletion of the gene product should always be performed.^40^ We have here shown through copy number analysis and deep targeted sequencing of cDNA that the RNA‐products are nonfunctional. Cell culture was also performed for multiple months, making prevailing protein products extremely unlikely, especially for p53 which has a half‐life of merely 20 min.

It is also possible that prolonged culture could result in a form of clonal adaptation where the stable optimum for some cell populations is a diploid cell state. If this is true, the cells should be aneuploid in the initial stages, but eventually converge toward their diploid ancestor. In this study we did not see any aneuploid subclones for neither the *RB1* nor *CDKN2A* knocked‐out cell lines, despite sampling at multiple time points. For example, there may also be a difference between having a single cell with an *RB1* loss among WT cells compared to having an *RB1* depleted cell among cells similar to itself genetically, as in the present culture system. The cell–cell interaction and competition among clones will probably be different. Hence, the surrounding cells could impact the expansion of a mutated cell. Further studies of this should be performed in the future.

A limitation of the present study is the use of a single cell line. It is possible that different cell lines, despite having a diploid genome, exhibit different phenotypical profiles, and thus begin at different positions in the epigenetic landscape. Hence, knocking out the same gene in different cell lines, could potentially cause diverse outcomes. The cell line used in this study was although shown to be karyotypically diploid and both genetically and phenotypically stable despite passaging for up to 20 weeks. Knocking out *RB1*, *CDKN2A*, and *TP53* respectively caused phenotypical changes in all replicates, while only the *TP53*‐knocked out cells managed to maintain surviving detectable clones with copy number alterations. Thus, there was both a clear impact on the transcriptomic profile after knock‐out of these genes in this cell line compared to controls, as well as a clear difference between the genotype between individual knock‐outs. In future studies, the impact of knock‐out of *TP53*, *RB1*, and *CDKN2A*, respectively, in multiple different cell lines could reveal potential cell line specific characteristics.

The present study also stresses the impact of clonal evolution in in vitro settings. Even the ancestral Bj‐5ta cell line showed an extensive subclonal evolution with an entirely new clone taking over the sample after prolonged passaging. This was replicated in two different laboratories with parallel cultures of the same cell line.^29^ Interestingly the cells that had been knocked out for *RB1* or *CDKN2A* exhibited less genomic alterations than the wild type cells cultured at one of the sites. Hence, the manipulation of the cells by knocking out *RB1*, *CDKN2A*, and *TP53* managed to affect the inherent evolutionary trajectory of the cell line seen when culturing it without manipulation. This also stresses the need to consider the clonal evolution of the cell line itself when using them for research, to not impede the inferred results of the experiments.

In conclusion, using a very pure model system, the present study questions the long‐held notion that *RB1* and *CDKN2A* depleted cells exhibit CIN and puts them in stark contrast to *TP53*‐depleted cells.

## AUTHOR CONTRIBUTIONS

NA, LM, JK, JN, KHN and DG conceived and designed the project. LM and JK cultured the cells. LM and JN prepared the knockout cell lines. NA analyzed the whole‐genome genotyping data. NA and KHS analyzed the bulk RNA‐seq data. NA performed phylogenetic analysis. NA wrote the manuscript and KHS, LM, JN, JK, KHN and DG contributed to the manuscript and interpretation of the results.

## CONFLICT OF INTEREST

The authors do not have any competing interests to declare.

## Supporting information


**Figure S1** Fishplots for the cell line passaging Fishplots for (A) the WT Bj5‐ta cells cultured at Lund university and (B) the WT Bj5‐ta cells cultured at Ben Gurion University in Israel.^29^ Above the fishplots the sample name and at which passage the corresponding sample was taken, is denoted. Below the fishplot there is a matrix where each column is a sample, each row a genetic alteration and the matrix elements is the MCF of that alteration in that particular sample.

**Figure S2** Fishplots for the knock‐out passaging Fishplots for (A) *RB1*, (B) *CDKN2A*, and (C) *TP53*. Above the fishplots the sample name and at which passage the corresponding sample was taken, is denoted. Below the fishplot there is a matrix where each column is a sample, each row a genetic alteration and the matrix elements is the MCF of that alteration in that particular sample.

**Figure S3** (**A)** A clonal map only containing the pie charts from Figure 1C. The arrows indicate increasing passaging time. (B**)** G‐banding of the *TP53* knocked‐out cells reveals a whole genome duplication in these cells. (C**)** The topmost graph is a SNP‐array profile for chromosome 6 centered on a diploid genome. Definitions from this is visualized with the log_2_ ratio. The proximal part of 6q (red square) in the *TP53* knocked‐out cells exhibits a complicated profile with gains and losses of the chromosomal segments. In the segment file (Dataset 1) this alteration is merely annotated as complex since it is not possible to decipher the evolutionary trajectory of this particular segment.

**Figure S4** Circos plots. Circos plots depicting segmental aberrations detected in the knockouts. (A) *RB1*, (B) *CDKN2A*, (C) WT Lund, (D) WT BGU, and (E) *TP53*. The innermost circle represents the first sampling point and the outermost circle the latest sample. The distribution of copy number alterations across the genome is visualized and the copy number is color coded from losses being blue and gains being red (Dataset 1).

**Figure S5** (A) MA‐plots showing, for each comparison, the mean fold change against the average expression for each gene before versus after shrinkage applied using the lfcShrink function with adaptive Student's *t* prior shrinkage estimator from the apeglm package (v.1.14.0), to reduce the number of false positives. (B**)** PCA‐plots based on rlog (regularized log) transformed raw RNA‐seq counts. The three first principal components are shown in this graph, colored by group and passaging time.

**Dataset S1:** All samples included in this study with specification of the passaging time they were harvested. The next sheets include the segment file, the event matrix and an overview matrix illustrating each genetic alteration's MCF across samples as well as the p‐ and W‐values for the comparison of the number of CNV events and CNV events >1 Mbp between the samples as well as the event matrices used for fish plot generation.

**Dataset S2:** The complete results tables obtained from DESeq2.

**Dataset S3:** All significantly enriched GO‐terms and KEGG‐pathways for the WT, *RB1*, *CDKN2A*, *TP53*, and EV samples.

**Appendix S1:** Supporting information.

## Data Availability

The data that supports the findings of this study are available in the supplementary material of this article.
