# Supplementary material for: Inactivation of RB1 , CDKN2A , and TP53 have distinct effects on genomic stability at side‐by‐side comparison in karyotypically normal cells
Source: Genes Chromosomes Cancer. 2022 Sep 30;62(2):93–100. doi: 10.1002/gcc.23096 (PMC10091693; doi:10.1002/gcc.23096)
Supplement: Supplementary file 1 — Figure S1 Fishplots for the cell line passaging Fishplots for (A) the WT Bj5‐ta cells cultured at Lund university and (B) the WT Bj5‐ta cells cultured at Ben Gurion University in Israel. 29 Above the fishplots the sample name and at which passage the corresponding sample was taken, is denoted. Below the fishplot there is a matrix where each column is a sample, each row a genetic alteration and the matrix elements is the MCF of that alteration in that particular sample. [file GCC-62-93-s002.pdf]

Supplementary Figure 1

a WT - LU

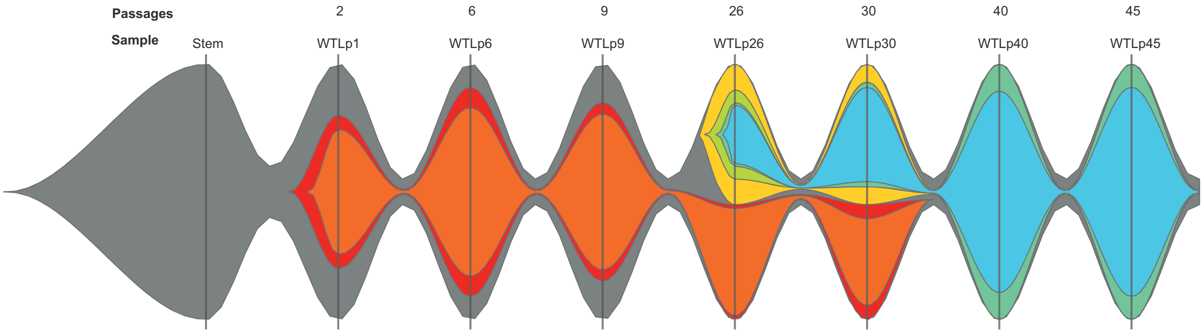

	Stem	WTLp2	WTLp6	WTLp9	WTLp26	WTLp30	WTL040	WTLp45
7 q33-q34 Gain (2+1)	100	100	100	100	100	100	100	100
8 p11.22-p11.22 Loss (1+0)	100	100	100	100	100	100	100	100
12 q23.1-q23.1 Gain (2+1)	100	100	100	100	100	100	100	100
19 p12-p12 Loss (1+0)	100	100	100	100	100	100	100	100
4 q13.2-q21.23 Loss (1+0)	0	60	82	70	45	45	0	0
2 p24.1-p24.1 Gain (3+1)	0	60	82	70	45	45	0	0
1 q43-q44 Gain (2+1)	0	49	66	61	42	34	0	0
4 p16.1-p16.1 Loss (1+0)	0	0	0	0	55	55	100	100
3 q13.31-q13.31 Loss (1+0) v2	0	0	0	0	35	41	100	100
14 q24.2-q32.33 Loss (1+0)	0	0	0	0	25	41	100	100
14 q11.2-q24.2 Gain (2+1)	0	0	0	0	23	37	79	82

b WT - BGU

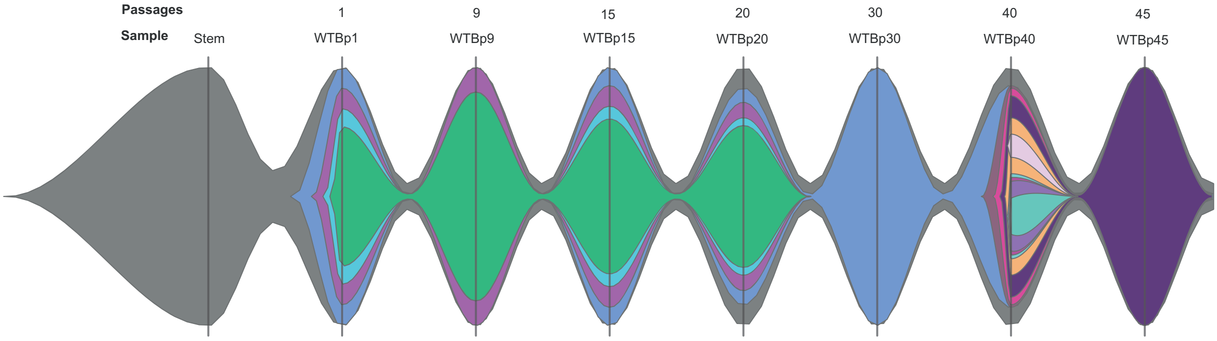

	Stem	WTBp1	WTBp9	WTBp15	WTBp20	WTBp30	WTBp40	WTBp45
7 q33-q34 Gain (2+1)	100	100	100	100	100	100	100	100
12 q23.1-q23.1 Gain (2+1)	100	100	100	100	100	100	100	100
19 p12-p12 Loss (1+0)	100	100	100	100	100	100	100	100
8 p11.22-p11.22 Loss (1+0)	0	100	100	100	84	100	86	100
2 p24.1-p24.1 Gain (3+1)	0	84	100	86	73	0	0	0
4 q13.2-q21.23 Loss (1+0)	0	84	100	86	73	0	0	0
1 q43-q44 Gain (2+1)	0	68	81	70	61	0	0	0
5 p15.2-p15.2 Gain (3+1)	0	54	81	60	55	0	0	0
4 p16.1-p16.1 Loss (1+0)	0	0	0	0	0	0	86	100
8 q22.2-q22.2 Loss (1+0)	0	0	0	0	0	0	84	100
3 q13.31-q13.31 Loss (1+0) v1	0	0	0	0	0	0	78	100
17 p13.3-p11.2 Loss (2+0)	0	0	0	0	0	0	61	0
9 q21.11-q22.33 Loss (1+0)	0	0	0	0	0	0	33	0
13 q11-q34 Loss (1+0)	0	0	0	0	0	0	30	0
6 q22.31-q27 Loss (1+0)	0	0	0	0	0	0	30	0
15 q11.2-q26.3 Loss (1+0) Whole	0	0	0	0	0	0	30	0
9 p24.3-p13.1 Loss (1+0) P-arm	0	0	0	0	0	0	27	0
2 p25.3-q37.3 Loss (1+0) Whole	0	0	0	0	0	0	14	0
1 q21.1-q44 Loss (1+0)	0	0	0	0	0	0	9	0
3 q25.32-q25.32 Loss (1+0)	0	0	0	0	0	0	0	0
