# Supplementary material for: Inactivation of RB1 , CDKN2A , and TP53 have distinct effects on genomic stability at side‐by‐side comparison in karyotypically normal cells
Source: Genes Chromosomes Cancer. 2022 Sep 30;62(2):93–100. doi: 10.1002/gcc.23096 (PMC10091693; doi:10.1002/gcc.23096)
Supplement: Supplementary file 2 — Figure S2 Fishplots for the knock‐out passaging Fishplots for (A) RB1, (B) CDKN2A, and (C) TP53. Above the fishplots the sample name and at which passage the corresponding sample was taken, is denoted. Below the fishplot there is a matrix where each column is a sample, each row a genetic alteration and the matrix elements is the MCF of that alteration in that particular sample. [file GCC-62-93-s003.pdf]

Supplementary Figure 2

a *RB1*

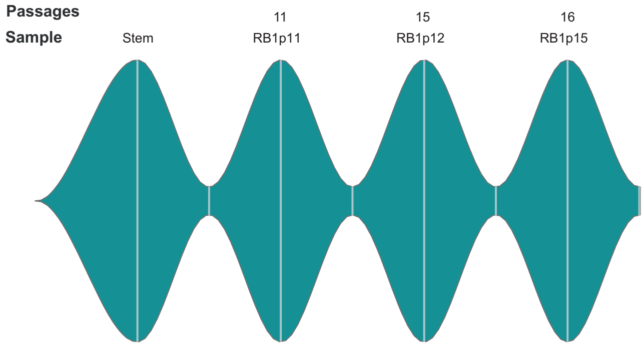

	Stem	RB1p11	RB1p12	RB1p15
7 q33-q34 Gain (2+1)	100	100	100	100
12 q23.1-q23.1 Gain (2+1)	100	100	100	100
8 p11.22-p11.22 Loss (1+0)	100	100	100	100
19 p12-p12 Loss (1+0)	100	100	100	100
3 q25.32-q25.32 Loss (1+0)	100	100	100	100
16 q23.1-q23.1 Gain (2+1)	100	100	100	100

b *CDKN2A*

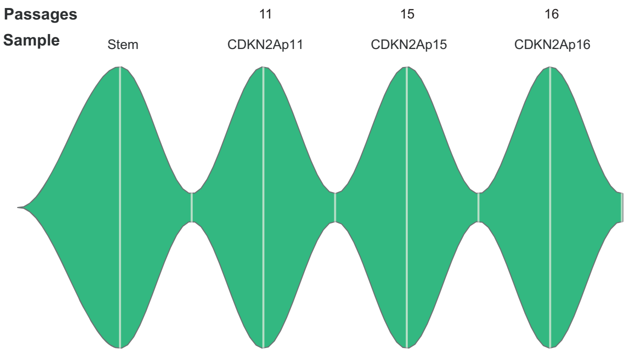

	Stem	CDKN2Ap11	CDKN2Ap12	CDKN2Ap15
7 q33-q34 Gain (2+1)	100	100	100	100
12 q23.1-q23.1 Gain (2+1)	100	100	100	100
8 p11.22-p11.22 Loss (1+0)	100	100	100	100
19 p12-p12 Loss (1+0)	100	100	100	100
7 q21.11-q21.11 Loss (1+0)	100	100	100	100
9 p22.2-p22.2 Gain (2+1)	100	100	100	100

c *TP53*

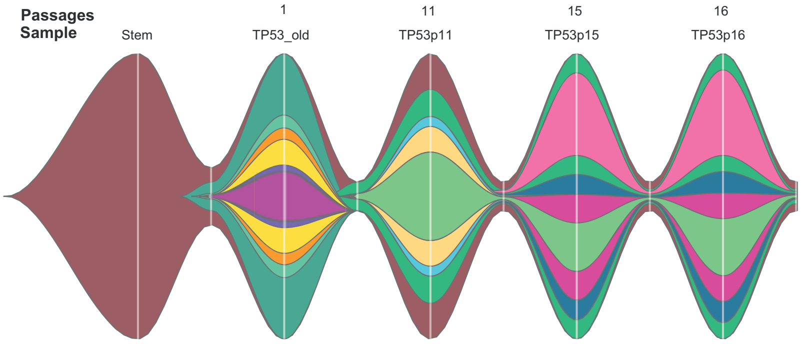

	Stem	TP53_old	TP53p11	TP53p15	TP53p16
7 q33-q34 Gain (2+1)	100	100	100	100	100
12 q23.1-q23.1 Gain (2+1)	100	100	100	100	100
8 p11.22-p11.22 Loss (1+0)	100	100	100	100	100
19 p12-p12 Loss (1+0)	100	100	100	100	100
3 q25.32-q25.32 Loss (1+0)	100	100	100	100	100
16 q23.1-q23.1 Gain (2+1)	100	100	100	100	100
1 K3 WGD	100	100	100	100	100
Complex	100	100	100	100	100
3 p26.3-p14.1 Loss (2+1)	100	100	100	100	100
6 q16.1-q27 Loss (2+1)	100	100	100	100	100
17 p13.3-p13.1 Loss (2+0)	100	100	100	100	100
3 p12.3-p12.3 Gain (3+2)	100	100	100	100	100
3 p14.1-p13 Gain (3+2)	0	100	0	0	0
9 p24.3-q34.3 Loss (2+1) Whole	0	100	0	0	0
17 q12-q25.3 Gain (3+2)	0	100	0	0	0
21 q11.2-q22.3 Loss (2+1) Whole	0	100	0	0	0
13 q12.12-q34 Loss (2+1) Whole	0	57	0	0	0
6 p25.3-p11.1 Loss (2+1) P-arm	0	57	0	0	0
2 q36.3-q37.3 Loss (2+1) v1	0	48	0	0	0
16 p13.3-p11.1 Gain (3+2) P-arm	0	40	0	0	0
7 p22.3-p11.1 Loss (2+1) P-arm	0	22	0	0	0
8 p23.3-q24.3 Gain (3+3) Whole	0	17	0	0	0
13 q12.12-q34 Loss (2+1) Whole	0	0	75	100	100
6 p25.3-p11.1 Loss (2+1) P-arm	0	0	56	51	53
2 p25.3-p11.2 Loss (2+1) P-arm	0	0	56	51	53
4 p16.3-q35.2 Loss (2+1) Whole	0	0	56	51	53
6 q14.1-q16.1 Loss (2+1)	0	0	56	51	53
7 p22.3-q36.3 Loss (2+1) Whole	0	0	56	51	53
9 p24.3-q34.3 Loss (1+1) Whole	0	0	56	51	53
21 q11.2-q22.3 Loss (1+0) Whole	0	0	56	51	53
3 p13-q29 Loss (2+1)	0	0	56	37	38
17 p12-p12 Loss (2+1)	0	0	49	37	38
17 q11.1-q25.3 Gain (3+2) Q-arm	0	0	31	37	38
22 q11.1-q13.33 Loss (2+1) Whole	0	0	31	17	20
2 q36.3-q37.3 Loss (2+1)	0	0	0	29	30
