# Supplementary figures and images for: Inactivation of RB1 , CDKN2A , and TP53 have distinct effects on genomic stability at side‐by‐side comparison in karyotypically normal cells
Source: Genes Chromosomes Cancer. 2022 Sep 30;62(2):93–100. doi: 10.1002/gcc.23096 (PMC10091693; doi:10.1002/gcc.23096)

Supplementary Figure 3

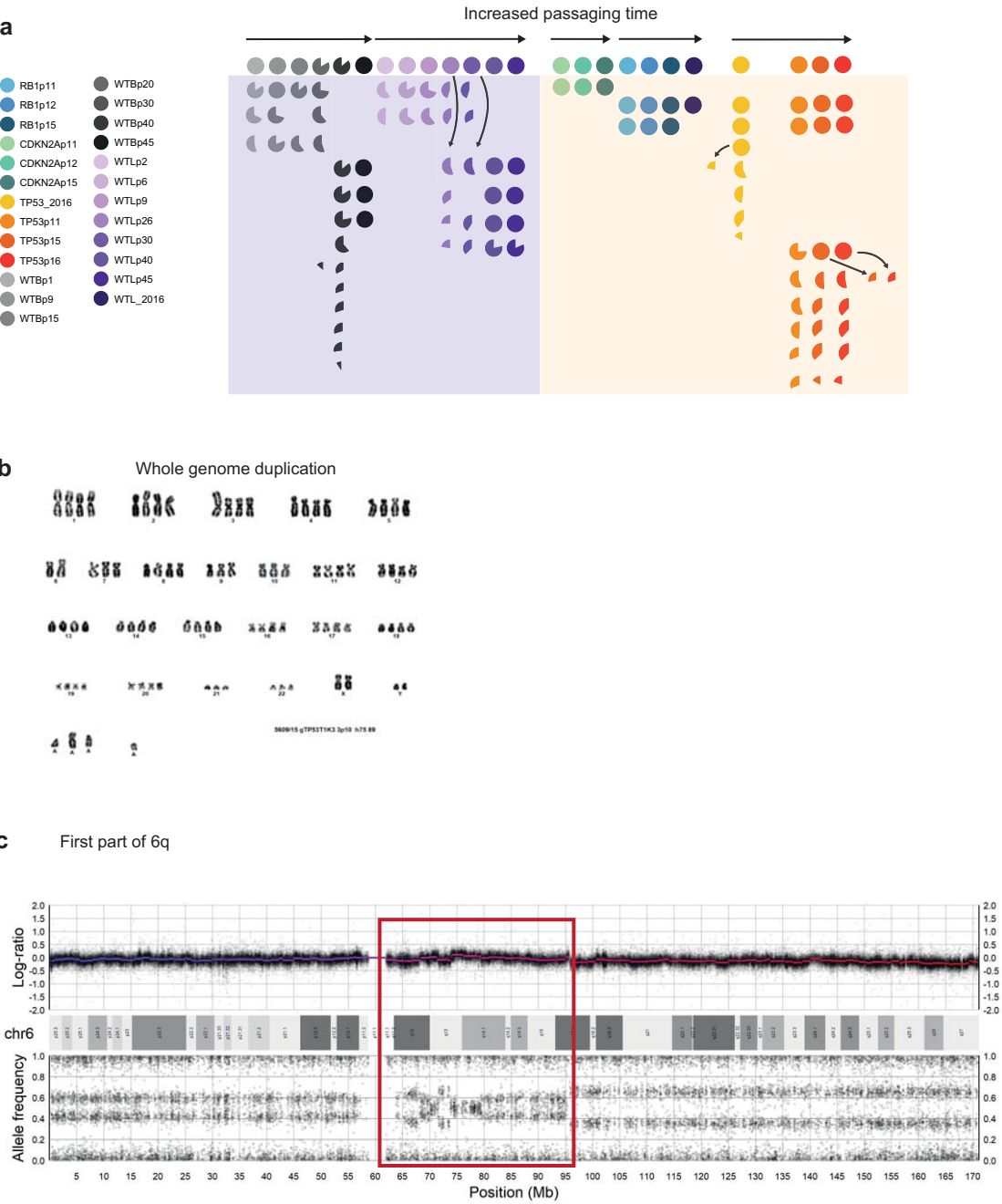

Supplement: Supplementary file 3 — Figure S3 (A) A clonal map only containing the pie charts from Figure 1C. The arrows indicate increasing passaging time. (B) G‐banding of the TP53 knocked‐out cells reveals a whole genome duplication in these cells. (C) The topmost graph is a SNP‐array profile for chromosome 6 centered on a diploid genome. Definitions from this is visualized with the log2 ratio. The proximal part of 6q (red square) in the TP53 knocked‐out cells exhibits a complicated profile with gains and losses of the chromosomal segments. In the segment file (Dataset 1) this alteration is merely annotated as complex since it is not possible to decipher the evolutionary trajectory of this particular segment. [file GCC-62-93-s009.pdf]

Supplementary Figure 4

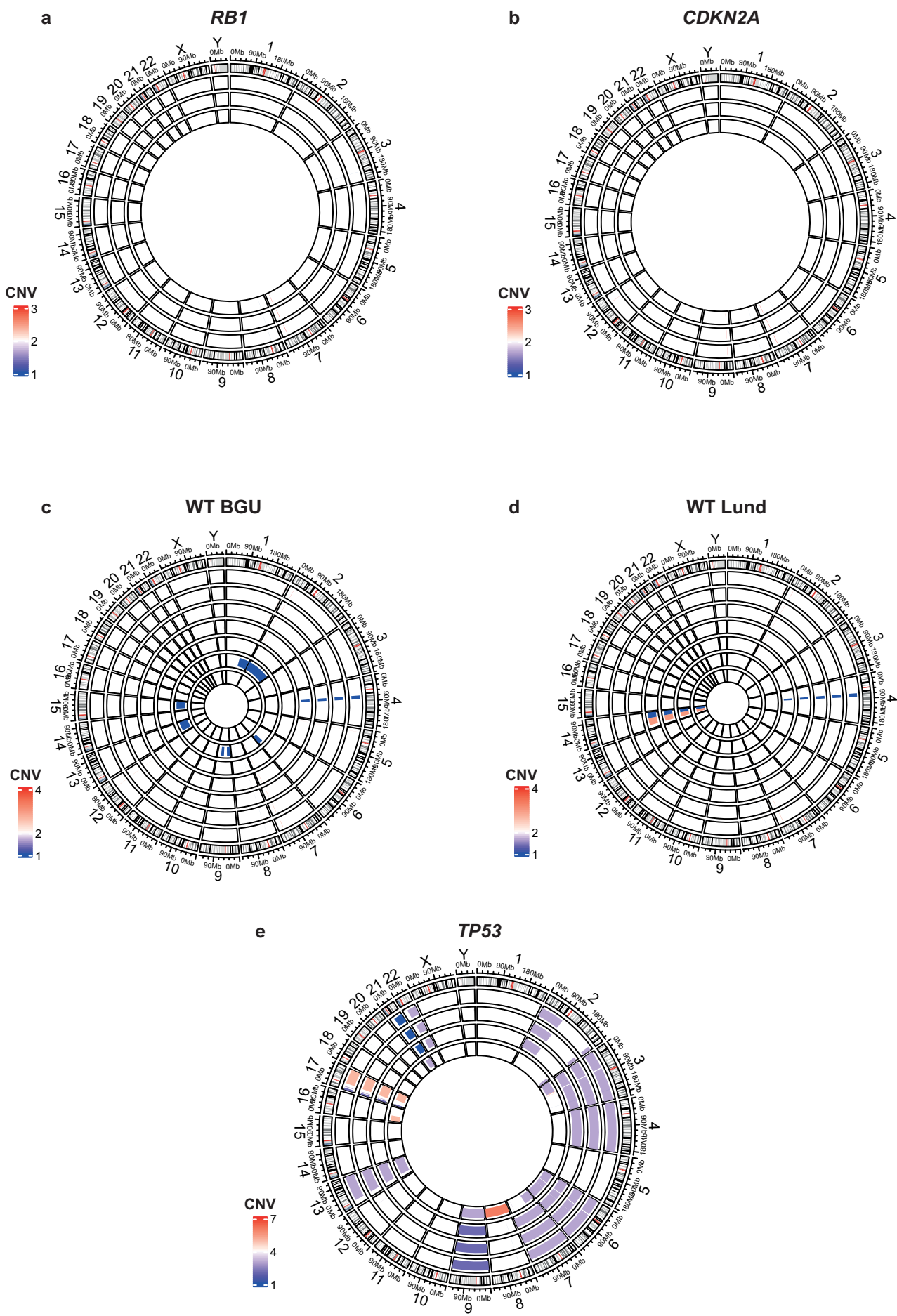

Supplement: Supplementary file 4 — Figure S4 Circos plots. Circos plots depicting segmental aberrations detected in the knockouts. (A) RB1, (B) CDKN2A, (C) WT Lund, (D) WT BGU, and (E) TP53. The innermost circle represents the first sampling point and the outermost circle the latest sample. The distribution of copy number alterations across the genome is visualized and the copy number is color coded from losses being blue and gains being red (Dataset 1). [file GCC-62-93-s008.pdf]

Supplementary Figure 5

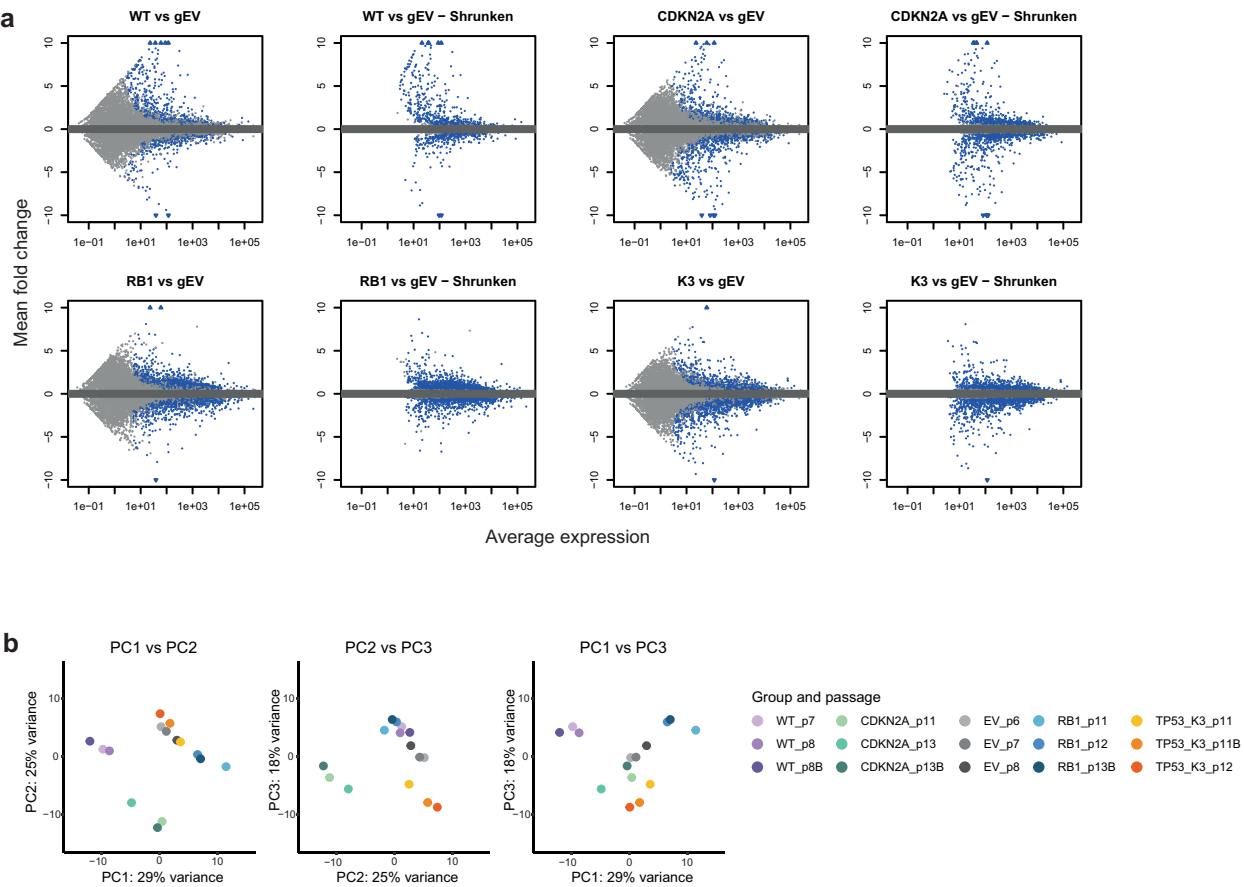

Supplement: Supplementary file 5 — Figure S5 (A) MA‐plots showing, for each comparison, the mean fold change against the average expression for each gene before versus after shrinkage applied using the lfcShrink function with adaptive Student's t prior shrinkage estimator from the apeglm package (v.1.14.0), to reduce the number of false positives. (B) PCA‐plots based on rlog (regularized log) transformed raw RNA‐seq counts. The three first principal components are shown in this graph, colored by group and passaging time. [file GCC-62-93-s005.pdf]
