# Supplementary material for: Inactivation of RB1 , CDKN2A , and TP53 have distinct effects on genomic stability at side‐by‐side comparison in karyotypically normal cells
Source: Genes Chromosomes Cancer. 2022 Sep 30;62(2):93–100. doi: 10.1002/gcc.23096 (PMC10091693; doi:10.1002/gcc.23096)
Supplement: Supplementary file 9 — Appendix S1: Supporting information. [file GCC-62-93-s001.pdf]

Supplementary methods

CRISPR-Cas9-mediated knockout and cell culture conditions

The cell line used in this study was an TERT-immortalized human foreskin fibroblast cell line (BJ-5ta cells; ATCC, LGC Standards, Middlesex, UK, CRL-4001). Wild type (WT) Bj-5ta was cultured in 4 parts DMEM, 1 part Medium 199 with the supplements 10% fetal bovine serum (FBS) and, 0.01 mg/ml hygromycin B. For the CRISPR-knocked cells 4 parts DMEM and 1 part Medium 199 was used with the supplements 10% FBS, 0.01 mg/ml hygromycin B, 5µg/ml blasticidin and, 0.5µg/ml puromycin. Details of growth conditions and how the CRISPR-mediated knockout was performed have been described previously for *TP53*[1]. To certify complete knockout of the respective gene, resistance-selected cells were single cell cloned followed by mutation screening with the Surveyor mutation detection kit (Integrated DNA Technologies, Inc., Coralville, IA). Clones with detected mutations were validated for homozygous or compound heterozygous mutations with Sanger Sequencing or Nextera sequencing (Illumina). An overview of the different experiments including time points for DNA and RNA extraction are presented in **Figure 1a** and **Supplementary Dataset 1**. DNA and RNA were extracted according to standard procedures as previously described[2].

RNA sequencing

Extracted RNA was prepared for sequencing using the TruSeq Stranded mRNA Library Prep (Illumina) according to the manufacturer's protocol, with the modification of the "Enrich DNA Fragment" step from 15 to 13 PCR cycles. Paired-end 150 base pair reads were generated from libraries using an Illumina NovaSeq 6000 instrument. Sequencing reads were aligned to the GRCh37/hg19 build using STAR v 2.5.2b[3]. For global gene expression analysis, read quantification was carried out using RSEM v 1.2.30[4].

In the *TP53* knockout cells, SNP array and Nextera deep sequencing analyses verified a deletion of large parts of chromosome arm 17p, including the *TP53* gene, accompanied by an additional deletion of 19 bp *TP53* exon 6 in the remaining allele. The mutation status of the *RB1* and *CDKN2A* clones was verified by Sanger and Nextera sequencing, both confirming an insertion of 1 bp in *RB1* and a 91 bp deletion in exon 2 in *CDKN2A* (hence, knocking out both p14/ARF and p16/INK4a), respectively. No normal allele was detected in any knockout clone.

Copy number profiling

DNA extracted from the Bj5-ta cell line samples were subjected to whole genome genotyping using the Cytoscan HD array (Thermo scientific) SNP array platform according to standard methods. Only clearly

visible genetic alterations in Chromosome Analysis Suite (ChAS), ≥ 50 kBp in size and with a marker count ≥ 50 were included in further analysis. Constitutional copy number variants were omitted based on manual comparison against the Database of Genomic Variants with hg19 as a reference genome. Information about each genetic alternation's genomic location, logarithmic median probe intensity ratio ($\log_2 R$) and type of aberration (loss or gain) were extracted. The function rawcopy[5] (v.1.1) in R was used on the Affymetrix fluorescence array intensity (CEL) files to normalize and estimate the $\log_2 R$ and B-allele frequency (BAF). These data were employed to generate Tumor Aberration Prediction Suite[6] (TAPS, v.2.0) plots visualizing copy number clusters for each chromosome and sample.

Clone size estimation

For copy number imbalances, the $\log_2 R_{ChAS}$-values together with the allelic composition of the genetic alteration was used in order to estimate the mutated sample fraction (MSF), defined as the percentage of cells in the sample harboring each particular genetic alteration.

$$\mathrm{MSF} = \frac{N_p \cdot 2^{\log_2 R_{ChAS}} - N_p}{N_t - N_p}$$

where N_p is the number of alleles of the background cells i.e., the ploidy level to which the $\log_2 R_{ChAS}$ has been normalized and N_t is the number of alleles of the genetic alteration in question.

For copy number neutral imbalances, BAF together with information on the allelic composition was used for MSF computation

$$\mathrm{MSF} = \frac{1 - 2 \cdot \mathrm{mBAF}}{\mathrm{mBAF}(N_A + N_B - 2) - N_B + 1}$$

where mBAF is the mirrored BAF, N_A is the number of A-alleles and N_B is the number of B-alleles and $N_B > N_A$.

The mutated clone fraction i.e., the proportion of non-normal cells harboring a specific genetic alteration was computed by

$$\mathrm{MCF} = \frac{\mathrm{MSF}}{\mathrm{TCF}}$$

where TCF is the tumor cell fraction. TCF was determined by extracting genetic alterations that were clearly clonal according to TAPS. The mean of these alterations' MSF values was determined as the TCF to which all genetic alterations were normalized. The interval of clonal events was computed as

$$\text{Interval of clonal events} = \frac{\mathrm{TCF} \pm 2 \cdot \mathrm{SD}_{MSF}}{\mathrm{TCF}}$$

where SD_{MSF} is the standard deviation of the MSF-values used to compute the TCF in the respective sample. Genetic alterations with a MCF within this interval were determined to be clonal and their MCF set to 100 %. The remaining genetic alterations were hence determined to be subclonal, and their MCF values were kept unchanged.

Phylogenetic reconstruction

To deduce the most probable evolutionary trajectory of the copy number alterations across the samples, the DEVOLUTION algorithm was used[7]. It takes an $u\ x\ v$ dimensional matrix as input (**Supplementary dataset 1**), containing information about each genetic alteration's chromosomal position, type (gain, loss, copy number neutral imbalance (cnni)), and its MCF across samples. This generated an event matrix of the subclones present across the samples along with information about the proportion of cells in each sample harboring the genotype in an event matrix (**Supplementary dataset 1**). The event matrix was used for phylogenetic reconstruction using the maximum likelihood and maximum parsimony methods separately, employing the R package phangorn (v2.8.1)[8] and visualized using the ggplot2 (v. 3.3.5) package.

Analysis of differentially expressed genes

Tximport (v.1.20.0) was used to import the matrix containing the expected counts from RSEM into the R environment. Each row represents one gene, the columns each sample and, the matrix elements are the expected count for each gene across samples. Since counts for transcripts in RNA-seq data may contain many rows with only zeros with no amount of gene expression, these were removed to reduce the size of the data object and to increase computational speed. Genes with zero length and zero abundance were therefore removed. This resulted in 5236 rows being removed from the data set, leaving 52584 rows. A design matrix was created incorporating the names of the samples, and to which group each sample belong (either WT, gEV, *RB1*, *CDKN2A* or *TP53*). The gEV samples are empty vector samples that are subjected to CRISPR-Cas9 without gRNA against any specific gene.

The R package DESeq2 (v.1.32.0, R version 4.1.1) was used to detect differentially expressed genes. The function DESeqDataSetFromTximport in the DESeq2 package was used to create the DESEQDataSet-object using the filtered expected counts matrix as input and the design matrix. The reference level was chosen using the function relevel to be the empty vector (gEV) samples. Subsequently, the DESeq function was applied to the DESEQDataSet-object. An additional filtering of the data was made on the output of this function, only keeping rows with a rowSum > 1, resulting in the removal of 28085 rows, leaving 24499 rows for further analysis. Rows with a row count sum of merely ≤ 1 are unlikely to produce significant results and is excluded either way due to the automatic

independent filtering, now included in the DESeq2 function, to increase the power. The analysis was performed on all samples at once, rather than subsetting the data and normalizing the subsets independently since this would result in different gene dispersion and sample size factors for each comparison, which are used when normalizing the raw counts and determining differential expression.

Log-fold change shrinkage was applied using the lfcShrink function with adaptive Student's t prior shrinkage estimator from the apeglm package (v.1.14.0), to reduce the number of false positives, yielding a final result matrix for the data set including information about the LFC and p-values for each gene in this sample compared to gEV. One data set was obtained for each comparison (RB1vsEV, CDKN2AvsEV, WTvsEV and TP53vsEV). LogFC before and after shrinkage was visualized using MA-plots. Genes were considered statistically significantly expressed if the adjusted p-value was below 0.05 after employing the Benjamini-Hochberg method, in order to further minimize the number of false positives.

For visualization, DESeq2's regularized log transformation (rlog) was used on the deseq-object with the option blind=FALSE, resulting in a matrix of regularized counts for data visualization. These values were used in order to produce the principal component analysis (PCA) plots. The principal components were calculated using the prcomp-function. The percentage of variation explained by each component was then computed using for each component x

$$\text{Variation explained by principal component x (\%)} = 100 \cdot \frac{\text{SD}_{\text{principal component x}}}{\text{sum}(\text{SD}^2)}$$

Gene set enrichment analysis of GO terms and KEGG pathways

The DESeq analysis resulted in a list of logFC-values for each gene for each group of three replicates compared to the empty vector samples. The R package clusterProfiler (v.4.0.5) was used to perform gene set enrichment analysis[9]. First, gseaGO was used to assess enrichment of gene ontology terms and gseaKEGG to assess enrichment of KEGG pathways. The analysis was performed on the entire gene list from the DESeq2 result table. As organism database, org.Hg,sgd.db was used. A p-value below 0.05 was considered a significant enrichment. The reported p-value for the gseaGO was further adjusted using the Benjamini-Hochberg procedure to correct for multiple testing.

Circos plots

Circos plots were produced using the segment files (Supplementary dataset 1) with the R package circlize[10].

1. Karlsson J, Valind A, Holmquist Mengelbier L, Bredin S, Cornmark L, Jansson C, et al. Four evolutionary trajectories underlie genetic intratumoral variation in childhood cancer. Nat Genet. 2018;50(7):944-50.
2. Saba KH, Cornmark L, Rissler M, Fioretos T, Astrom K, Haglund F, et al. Genetic profiling of a chondroblastoma-like osteosarcoma/malignant phosphaturic mesenchymal tumor of bone reveals a homozygous deletion of CDKN2A, intragenic deletion of DMD, and a targetable FN1-FGFR1 gene fusion. Genes Chromosomes Cancer. 2019;58(10):731-6.
3. Dobin A, Davis CA, Schlesinger F, Drenkow J, Zaleski C, Jha S, et al. STAR: ultrafast universal RNA-seq aligner. Bioinformatics. 2013;29(1):15-21.
4. Li B, Dewey CN. RSEM: accurate transcript quantification from RNA-Seq data with or without a reference genome. BMC Bioinformatics. 2011;12:323.
5. Mayrhofer M, Viklund B, Isaksson A. Rawcopy: Improved copy number analysis with Affymetrix arrays. Sci Rep. 2016;6:36158.
6. Rasmussen M, Sundstrom M, Goransson Kultima H, Botling J, Micke P, Birgisson H, et al. Allele-specific copy number analysis of tumor samples with aneuploidy and tumor heterogeneity. Genome biology. 2011;12(10):R108.
7. Andersson N, Chattopadhyay S, Valind A, Karlsson J, Gisselsson D. DEVOLUTION-A method for phylogenetic reconstruction of aneuploid cancers based on multiregional genotyping data. Commun Biol. 2021;4(1):1103.
8. Schliep KP. phangorn: phylogenetic analysis in R. Bioinformatics. 2011;27(4):592-3.
9. Wu T, Hu E, Xu S, Chen M, Guo P, Dai Z, et al. clusterProfiler 4.0: A universal enrichment tool for interpreting omics data. Innovation (N Y). 2021;2(3):100141.
10. Gu Z, Gu L, Eils R, Schlesner M, Brors B. circlize Implements and enhances circular visualization in R. Bioinformatics. 2014;30(19):2811-2.
